# Days at Home Following Hip Fracture Among Older Adults with Dementia

**DOI:** 10.1093/geroni/igaf122.2220

**Published:** 2025-12-31

**Authors:** Rebecca Rodin, Alexander Smith, Siqi Gan, John Boscardin, Edie Espejo, Lauren Hunt, Sean Morrison

**Affiliations:** Mount Sinai Medical Center, New York, New York, United States; University of California, San Francisco, San Francisco, California, United States; University of California, San Francisco, San Francisco, California, United States; University of California, San Francisco, San Francisco, California, United States; University of California, San Francisco, San Francisco, California, United States; University of California, San Francisco, San Francisco, California, United States; Icahn School of Medicine at Mount Sinai, New York, New York, United States

## Abstract

Hip fracture is common among older adults and nearly twice as common among those with dementia, who may be particularly vulnerable to their adverse effects. Time spent away from home may be an important indicator of such effects, but this outcome has not been previously examined in older adults with dementia (PWD) and hip fracture. We aimed to assess the impact of hip fracture hospitalization on days alive at home among community-dwelling PWD age 65+ compared to those without dementia utilizing 100% Medicare claims data (2012-2021). We did not report p values or confidence intervals due to their limited utility in very large sample sizes. We identified 1,853,295 older adults (mean age [SD] 82.5 [8.1]); 1,307,640 [70.6%] female) with a hip fracture hospitalization, of whom 556,765 (30.0%) had dementia. PWD had fewer days at home within 1-year post-hip fracture compared to those without dementia (adjusted mean 181 days vs. 267 days; difference 86 days), including more nursing home days (adjusted mean 37 days vs. 11 days; difference 26 days), skilled nursing facility days (adjusted mean 33 days vs. 24 days, difference 9 days), and days after death (adjusted mean 103 days vs. 51 days; difference 52 days). These findings indicate that older adults with dementia had 52 fewer days alive and spent >1 month more in long-term care or post-acute rehabilitation/skilled-nursing facilities in the year following hip fracture than those without dementia.

